# Prediction study of electric energy production in important power production base, China

**DOI:** 10.1038/s41598-022-25885-w

**Published:** 2022-12-12

**Authors:** XiXun Zhu, Zhixin Song, Gan Sen, Maozai Tian, Yanling Zheng, Bing Zhu

**Affiliations:** 1 Department of Computer Engineering, Jingchu University of Technology, Jingmen, 448000 Hubei, People’s Republic of China; 2 College of Medical Engineering and Technology, Xinjiang Medical University, Urumqi, 830011 People’s Republic of China; 3 Xinjiang Tianshan Cement Co. Ltd, Urumqi, 830013 People’s Republic of China

**Keywords:** Energy science and technology, Energy storage

## Abstract

Xinjiang is an important power production base in China, and its electric energy production needs not only meet the demand of Xinjiang's electricity consumption, but also make up for the shortage of electricity in at least 19 provinces or cities in China. Therefore, it is of great significance to know ahead of time the electric energy production of Xinjiang in the future. In such terms, accurate electric energy production forecasts are imperative for decision makers to develop an optimal strategy that includes not only risk reduction, but also the betterment of the economy and society as a whole. According to the characteristics of the historical data of monthly electricity generation in Xinjiang from January 2001 to August 2020 , the suitable and widely used SARIMA (Seasonal autoregressive integrated moving mean model) method and Holt-winter method were used to construct the monthly electric energy production in Xinjiang for the first time. The results of our analysis showed that the established SARIMA((1,2,3,4,6,7,11),2,1)(1,0,1)_12_ model had higher prediction accuracy than that of the established Holt-Winters' multiplicative model. We predicted the monthly electric energy production from August 2021 to August 2022 by the SARIMA((1,2,3,4,6,7,11),2,1)(1,0,1)_12_ model, and errors are very small compared to the actual values, indicating that our model has a very good prediction performance. Therefore, based on our study, we provided a simple and easy scientific tool for the future power output prediction in Xinjiang. Our research methods and research ideas can also provide scientific reference for the prediction of electric energy production elsewhere.

## Introduction

The invention and application of electric power set off the second high tide of industrialization. The large-scale electric power system appeared in the twentieth century is one of the most important achievements in the history of human engineering science. The rapid development of electric power industry has promoted economic development and social progress.

Xinjiang is an important power production base in China. Since the reform and opening-up, with the rapid economic development of Xinjiang, Xinjiang power industry has made great progress with the support of the central government and inland provinces of China. At present, the existing power generation methods are thermal power, hydropower, wind power and photovoltaic power generation, of which thermal power accounts for the largest proportion, followed by the order of hydropower, wind power, photovoltaic power generation. In today's society which emphasizes Green Environmental Protection and sustainable development, the government in Xinjiang has made great efforts to develop traditional and clean energy sources, promote green projects for the harmonious development of energy, the economy and the environment, and continue the trend of green and low-carbon development in power generation. In terms of the share of electricity generation over the years, the share of thermal power generation has been decreasing year by year, the share of hydropower, photovoltaic power generation and wind power has increased year by year.

In 2019, electric energy production in Xinjiang ranks in the forefront of many provinces and cities in China. Electric energy production in Xinjiang has not only met the demand of power consumption in Xinjiang, but also properly solved the problem of power shortage in some provinces and cities in China. According to statistics from Xinjiang Power Exchange Center Co., Ltd., in 2019, the “sending out electricity in Xinjiang” exceeded 71.2 billion kwh which was 1.4 times the size in 2018, and the power transmission range reached 19 provinces and municipalities (http://www.camchina.cn/sp/9680html). The electric power in Xinjiang not only plays an important role in ensuring the development of Xinjiang, but also plays an important role in some provinces and cities supplied power by Xinjiang, ensuring the healthy and coordinated development of the economy and people's life in these supplied areas.

From the perspective of changes in power consumption demand, with the continuous improvement of the level of social informatization, power will become the most important terminal consumption energy, and its status will continue to rise, and power consumption will continue to grow, especially with the coming of the information and Internet Age. The degree of electrification of the whole society is increasing, and the demand of electric power consumption is increasing obviously^1–3^. This also puts forward higher requirements for the power generation capacity of the power industry. Therefore, scientific forecasting of the electric energy production of Xinjiang is of great significance to the development planning of the power industry of Xinjiang. It can help Xinjiang and the provinces and cities supplied electricity by Xinjiang to accurately grasp the situation of power supply, make accurate predictions, and make good electricity demand arrangements in advance.

A common method of prediction is to establish an appropriate prediction model and make prediction analysis according to the characteristics of time series data. An important way to analyze time series is to study the statistical laws of the data generation patterns, and to assume that these laws will still play an important role in the future. Many mathematical models can be established to approximate this law and to make reasonable predictions for variables^4–6^. In the 1970s, the American scholar Box and the British statistician Jenkins cooperated with each other to develop a perfect statistical prediction method named Box-Jenkins method^5,6^. There are many models in this method: autoregressive model AR (p), moving average model MA (q), autoregressive moving average model ARMA (p, q), autoregressive integrated moving average model ARIMA (p,d,q), seasonal autoregressive integrated moving average model SARIMA (p,d,q)(P,D,Q) s, etc. All the first four models are special forms of SARIMA (p,d,q)(P,D,Q)s models. In above models, the p is the order of autoregression, the q is the order of moving average, the d is the times of ordinary difference when the time series becomes stationary, P is the order of seasonal autoregression, Q is the order of seasonal moving average, and D is seasonal difference times, and s is the seasonal cycle. Generally speaking, for the monthly time series, s is 12. In the analysis of time series prediction, we often need to use different models according to the characteristics of data changes. Because Box-Jenkins method can often obtain high prediction accuracy, they are widely used in time series prediction analysis in various fields^7,8^. Application of Box-Jenkins methods in non-energy forecasting: Ilie et al.^9^ pointed out that ARIMA models were suitable for making predictions during COVID-19 crisis and offered an idea of the COVID-19 epidemiological stage of Ukraine, Romania, the Republic of Moldova, Serbia, Bulgaria, Hungary, USA, Brazil, and India. Hernandez-Matamoros et al.^10^ applied ARIMA models to forecast the COVID19 of many regions successfully. He et al.^11^ found that the ARIMA model could effectively predict the positive rate of influenza virus in a short time in Wuhan, China. Fanoodi et al.^12^ pointed out the ARIMA models was more accurate in predicting the uncertainties in demand than the baseline model used in Zahedan Blood Transfusion Center. Zheng et al.^13^ used the ARIMA model to predict the total health expenditure in China from 1978 to 2022. Liu et al.^14^ found that the ARIMA model could be used to predict the seasonality and trend of pulmonary tuberculosis in the Chinese population. Keskin et al.^15^ applied ARIMA model to simulate total electron content, earthquake and radon relationship identification. Yingzi et al.^16^ applied ARIMA model to predict vehicle speed. Application of Box-Jenkins methods in energy forecasting: González-Romera et al.^17^ found that the ARIMA model could be used to predict the medium-term electric energy demand based on the Spanish monthly electric demand series. Parag et al.^18^ revealed that ARIMA (1,0,0)(0,1,1) model was the best fitted model for energy consumption and ARIMA (0,1,4)(0,1,1) was the best fitted model for greenhouse emission of a pig iron manufacturing organization of India. Aasim et al.^19^ put forward the ARIMA model for very short-term wind speed forecasting. Contreras et al.^20^ pointed out that ARIMA model was good to predict next-day electricity prices. Kavasseri et al.^21^ found that ARIMA models could forecast day-ahead wind speed well. Wang et al.^22^ did a good prediction for U.S. shale gas monthly production using a hybrid ARIMA and metabolic nonlinear grey model.

The exponential smoothing method is also a perfect statistical prediction method, which is widely used in forecasting research. According to the different times of smoothing, the exponential smoothing method is divided into: the single exponential smoothing method, the double exponential smoothing method and the triple exponential smoothing method^23^. The triple exponential smoothing model was developed by Holt and Winters, which is also called Holt-Winters method, it includes Holt-Winters' additive method and Holt-Winters' multiplicative methods. Liljana et al.^24^ found that Holt–Winters methods ensured the best forecasting values in purpose of long-term heat load forecasting and monthly short-term heat load forecasting of the Company Energetika Ljubljana in the Republic of Slovenia. Vincenzo et al.^25^ employed Holt–Winters exponential smoothing method for the nonresidential electricity consumption prediction in Romania, they found Holt–Winter’ prediction accuracy was good in relation to the time horizon considered in their study. Guan et al.^26^ developed Holt–Winters additive model and Holt–Winters multiplicative model for short-term extrapolation forecast based on monthly reported human brucellosis cases in mainland China. Zhang et al.^27^ found that Holt winter method could predict tuberculosis registration rates in Henan Province, China successfully^28–31^.

In this study, we carefully analyzed the trend of historical monthly electric energy production in Xinjiang. According to the characteristics of the data changes, we tried to build SARIMA model^4^, Holt-Winters' additive model and Holt-Winters' multiplicative model^5^ to do fitting analysis of Xinjiang monthly electricity generation. And then, we compared and analyzed the fitting and prediction precision of these established models. Finally, we applied the established model to do the prediction analysis of Xinjiang monthly power generation from August 2021 to December 2022. Our prediction results could provide a scientific reference for Xinjiang and some provinces and cities of needing Xinjiang electric power to do a good job in the allocation of power resources in advance. Our research methods can also provide research ideas for researchers to predict power production in other place.

## Data and methodology

### Data

**Figure 1:**
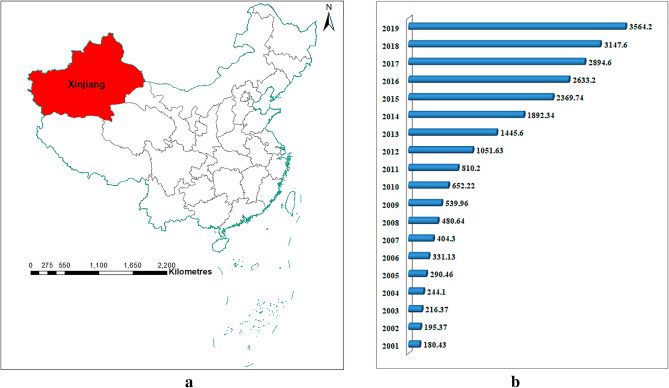
(**a**) The red area in the figure is the geographical location of Xinjiang, (this figure is plotted by ArcMap10.4); (**b**) Xinjiang annual electric energy production (billion kWh).

In this study, we focus on the prediction and analysis of Xinjiang's monthly electric energy production. We collected the data of Xinjiang's monthly electric energy production from January 2001 to August 2022, including 260 months' data, which are derived from the National Bureau of Statistics of China. Our research area and Xinjiang annual electric energy production data are shown in Fig. 1.

### Methodology

#### SARIMA model

SARIMA (seasonal autoregressive integrated moving average) model can well predict and analyze time series with seasonality, trend and randomness^4–6^. The SARIMA(p,d,q)(P,D,Q)s model can be expressed as follows:

1 $$\begin{aligned}{}&\Phi_{p} (L)A_{P} (L^{s} )\Delta^{d} \Delta_{s}^{D} y_{t} = \Theta_{q} (L^{{}} )B_{Q} (L^{s} )\varepsilon_{t},\nonumber\\& \Phi_{p} (L) = 1 - \varphi_{1} L - \varphi_{2} L - \cdots - \varphi_{p} L^{p}, \nonumber\\ & A_{P} (L^{s} ) = 1 - \alpha_{1} L^{s} - \alpha_{2} L^{2s} - \cdots - \alpha_{P} L^{Ps}, \nonumber\\ &\Theta_{q} (L) = 1 + \theta_{1} L + \theta_{2} L + \cdots + \theta_{q} L^{q},\nonumber \\ &B_{Q} (L^{s} ) = 1 + \beta_{1} L^{s} + \beta_{2} L^{2s} + \cdots + \beta_{Q} L^{Qs}, \nonumber\\ &\Delta_{s} y_{t} = (1 - L^{s} )y_{t} = y_{t} - y_{t - s},\nonumber \\ &\Delta_{s} = 1 - L^{s}, \nonumber\\& \varepsilon_{t} :WN(0,\sigma^{2} )\end{aligned}$$

where, $\Delta$ and $\Delta_{s}$ denote non-seasonal and seasonal differences, respectively.$\varphi ,\Phi ,\theta {\kern 1pt} {\kern 1pt} {\kern 1pt} {\text{and}}{\kern 1pt} {\kern 1pt} {\kern 1pt} \Theta$ are the parameters of the model, $\varepsilon_{t}$ is white noise with independent and identical distribution. A sparse coefficient model is a special case of SARIMA model. If some of the coefficients in the SARIMA model are 0, then, the model becomes a sparse coefficient model. If only the autoregressive part has some missing terms, the sparse coefficient model can be recorded as:SARIMA((p1,…,pm),d,q)(P,D,Q)s.

The construction of SARIMA model has main four steps:

**Step 1.** SARIMA model is built on the basis of stationary time series, so the stationarity of time series is an important prerequisite for modeling. The Augmented Dickey-Fuller (ADF) unit root test model can be used to test the stationary of time series (if p-value is less than 0.05, the data is stationary). If the time series is un-stationary, it can be stabilized by some operations, such as ordinary difference or seasonal difference.

**Step 2.** To draw the autocorrelation function (ACF) and partial autocorrelation function (PACF) of the smooth data, which can help to determine the possible values of P, Q, p, and q in the model.

**Step 3.** After determining p, q, P and Q values, it is necessary to check the parameters of the model for determining the values of p, q, P and Q, and calculate the R^2^, Akaike information criterion (AIC) and Schwarz criterion (SC) of the model. The bigger the R^2^ is, the smaller the AIC and SC are, the better the model is. The mathematical expressions of R^2^, AIC, and SC are as follows:

2 $$AIC = - 2\ln (L) + 2k.$$

3 $$SC = - 2\ln (L) + \ln (n) \times k.$$

4 $$R^{2} = 1 - {\frac{{\sum\nolimits_{i - 1}^{n} {(\hat{y}_{i} - y_{i} )^{2} } }}{{\sum\nolimits_{i}^{n} {(\overline{y}_{i} - y_{i} )^{2} } }}},$$

where, L is the maximum likelihood of the model, n is the number of observations, and k is the number of variables in the model.

**Step 4.** To plot ACF and PACF and do Box-Jenkins Q test of residuals to help judging whether or not model residuals are white noise. If the residuals are white noise, the autocorrelation coefficients and partial correlation coefficients of the residuals are basically within twice the standard deviation, and the p-value of Box-Jenkins Q test is greater than 0.05, which indicates that the model has good fitting performance and can be used for prediction analysis.

**Figure 2:**
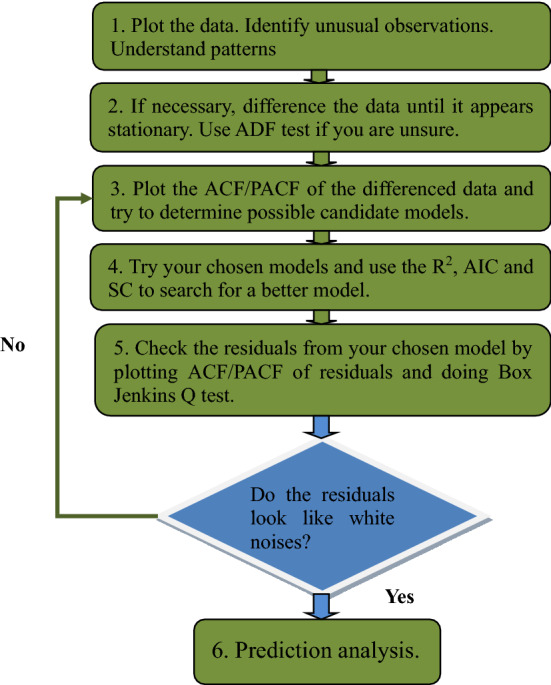
The modeling flowchart of SARIMA method.

To understand more intuitively the steps of SARIMA model building, we draw SARIMA flow chart Fig. 2.

#### Holt-Winters' method

Holt-Winters' method is generally more suitable for forecasting and analyzing time series with trend, seasonality and randomness.

Holt-Winters' additive model has the following expression^31–34^:

5 $$\begin{aligned} &\hat{y}_{{{{t + h} /t}}} = l_{t} + hb_{t} + s_{t - m + h}, \nonumber\\& l_{t} = \alpha (y_{t} - s_{t - m} ) + (1 - \alpha )(l_{t - 1} + b_{t - 1} ), \nonumber\\& b_{t} = \beta (l_{t} - l_{t - 1} ) + (1 - \beta )b_{t - 1}, \nonumber\\& s_{t} = \gamma (y_{t} - l_{t - 1} - b_{t - 1} ) + (1 - \gamma )s_{t - m} .\end{aligned}$$

Holt-Winters' multiplicative model has the following expression^31–34^:

6 $$\begin{aligned} &\hat{y}_{{{{t + h}/t}}} = (l_{t} + hb_{t} )s_{t - m + h}, \nonumber\\& l_{t} = \alpha \frac{{y_{t} }}{{s_{t - m} }} + (1 - \alpha )(l_{t - 1} + b_{t - 1} ), \nonumber\\& b_{t} = \beta (l_{t} - l_{t - 1} ) + (1 - \beta )b_{t - 1}, \nonumber\\& s_{t} = \gamma \frac{{y_{t} }}{{(l_{t - 1} + b_{t - 1} )}} + (1 - \gamma )s_{t - m} ,\end{aligned}$$

where, $0 \le \alpha \le 1$, $0 \le \beta \le 1$, $0 \le \gamma \le 1{ - }\alpha$ . $s_{t - m + h}$ is the seasonal term. *α*, *β*, and *γ* are the smoothing parameters. m is seasonal periods, and h is the predicted step size.

There are three main steps for Holt-Winters modeling process: first, to do model parameter estimation; Second, to do model fitting accuracy analysis, third, using the Box-Jenkins Q method and the normal distribution map of the residuals to test whether or not the residual data is white noise. If the test can pass, it shows that the model has good fitting performance, then, model can be used for prediction analysis.

#### The indexes for model comparison

Root mean square error (RMSE), mean absolute error (MAE), mean absolute percentage error (MAPE) are the measure indexes of the accuracy of model fitting, and they are widely used to compare the accuracy of model prediction. The smaller the three values, the higher the fitting accuracy, the better the model performance. In this study, these three indexes are used to compare the performance of SARIMA model and Holt-Winters model. where,

7 $$RMSE = \sqrt {\frac{1}{n}\sum\limits_{i = 1}^{n} {(y_{i} - \hat{y}_{i} )^{2} } } .$$

8 $$MAE = \frac{1}{n}\sum\limits_{i = 1}^{n} {{|}y_{i} - \hat{y}_{i} {|}} .$$

9 $$MAPE = \frac{1}{n}\sum\limits_{i = 1}^{n} {{|}\frac{{y_{i} - \hat{y}_{i} }}{{y_{i} }}{|}} \cdot 100.$$

#### Data analysis software

In the study, data were analyzed using ArcMap10.4, R3.6.2, and Eviews7.0.

## Results

**Figure 3:**
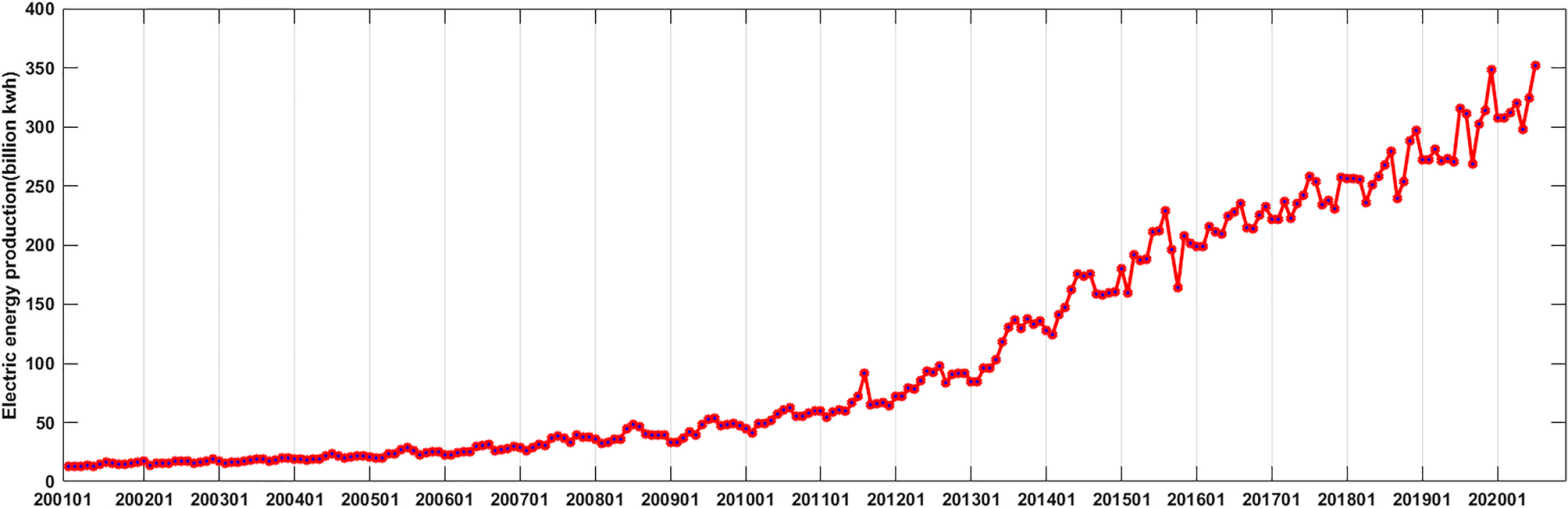
Time series of electric energy production in Xinjiang from January 2001 to July 2020 (since the x-axis length is limited, only the January location of each year is marked in the figure).

We divided the data into three parts; the data that was used for the modeling in this study are monthly electric energy production in Xinjiang from January 2001 to July 2020. Data from August 2020 to July 2021 were used to test the model prediction effect, and data from August 2021 to August 2022 were used to view the model prediction performance. The change diagram of the time series for modeling is shown in Fig. 3. It can be seen from the diagram that the time series has obvious trend and randomness. From 2001 to 2010, Xinjiang's electric energy production showed a slow growth trend. And from 2011 to 2020, it showed a rapid growth, and the fluctuation of monthly electric energy production increased.

### Modeling analysis of SARIMA model

The SARIMA model takes into account not only the dependence of economic phenomena on time series, but also the disturbance of stochastic fluctuation in the process of economic forecasting; it is one of the widely used methods in recent years.

**Figure 4:**
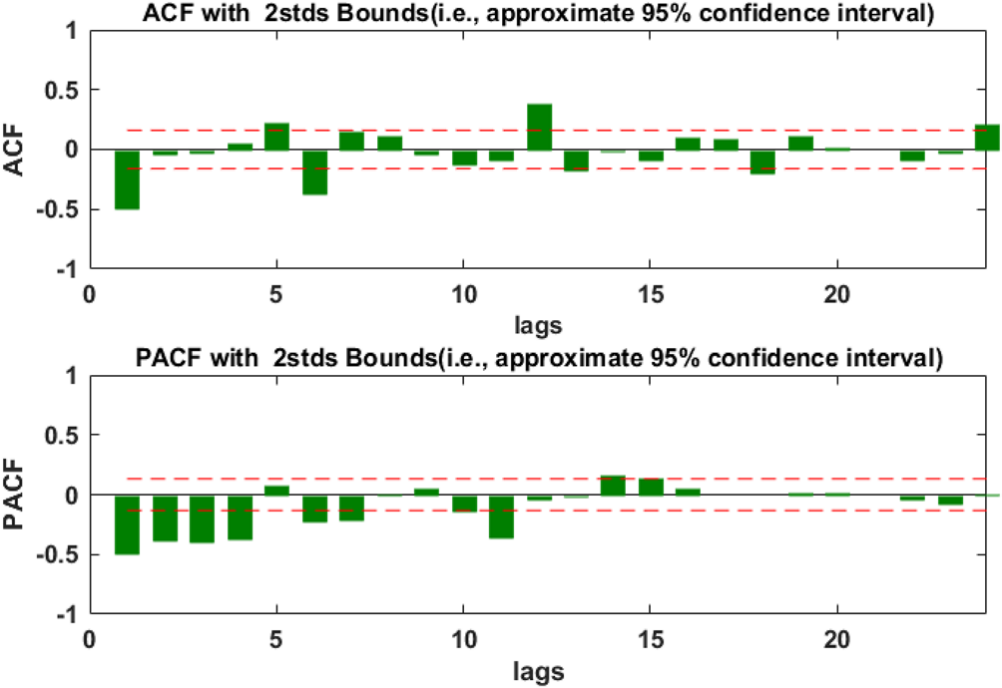
Autocorrelation and partial correlation diagram of electric energy production time series in Xinjiang after quadratic difference.

**Figure 5:**
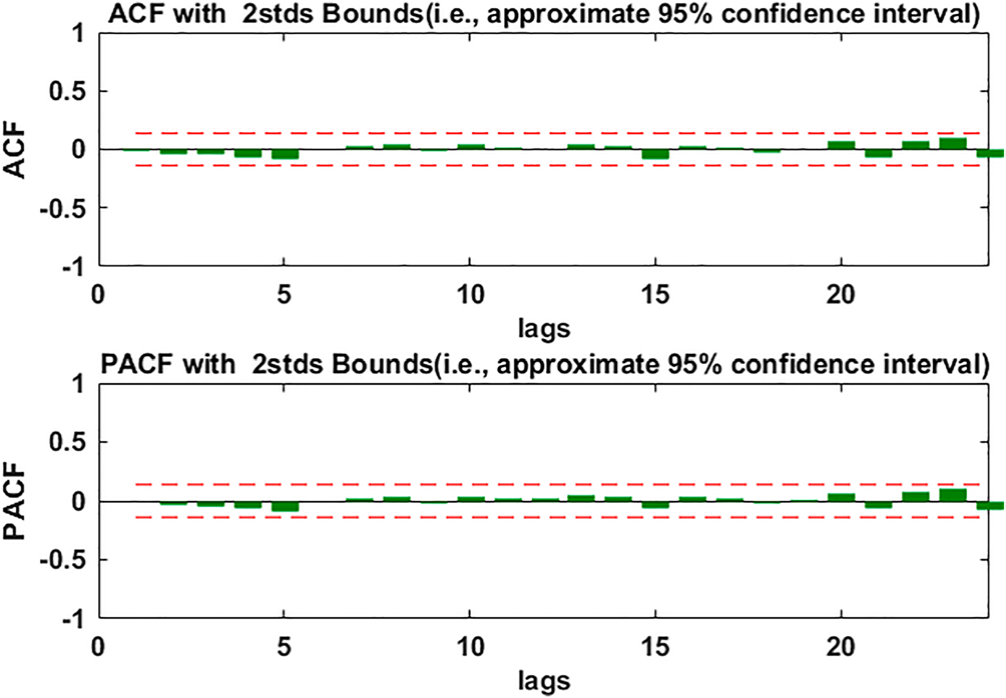
The ACF and PACF of SARIMA((1,2,3,4,6,7,11),2,1)(1,0,1)_12_ residuals.

During the construction of the SARIMA model, the data must be stationary, therefore, we first used ADF to test whether or not the time series from January 2001 to July 2020 was stationary. The test results showed that the p-value was greater than 0.05, which indicated that the original time series was not stationary, so, we did a common difference of data. The ADF test of the data after difference showed that the data was still not stationary. Then, we did the secondary ordinary difference of the data, the p-value of the ADF test of the data after the secondary ordinary difference was less than 0.05, this indicated that the data after the secondary difference was stationary (d = 2, D = 0). And the test results were shown in Table 1. To draw the ACF and PACF of stationary data (see Fig. 4), we could see these correlation coefficients of the data at lag 1, 5, 6, 12 and 24 were relatively large, so we let q take 1, 5 or 6,and Q take1. Because these partial correlation coefficients of the data at lag 1, 2, 3, 4, 6, 7, 11 and 12 were relatively large, so we let p take 1, 2, 3, 4, 6, 7 or 11, and P take1, s take 12. According to the combination of the values of p, q, P, Q, several SARIMA models were established and the parameters of the models were tested, and the R^2^, AIC and SC values of the model were calculated simultaneously. In the end, only six models passed the parameters test, and the results were shown in Table 2. The AIC and SC of the Model 1 were the smallest. We used the Box-Jenkins Q method to test whether or not the residual was white noise, and the p-value of the test was less than 0.05, which indicated that the correlation between the residuals was significant. Therefore, the residuals were not white noise, which showed that the model was not good enough to be used for prediction analysis. When comparing the R^2^, AIC and SC values of the remaining five models, it was found that the Model 6 had the largest R^2^ and the smallest AIC. The p-value of Box-Jenkins Q test of Model 6 was more than 0.05, which indicated that there was no correlation between model residues. Furthermore, the ACF and PACF of the residuals of Model 6 were plotted (see Fig. 5). The autocorrelation and partial correlation coefficients of the residuals were almost within twice the standard deviation, this further indicated that the residuals at each lag were not correlated and they were white noise, which indicated that Model 6 has a good fitting performance, and could capture original data information well. Therefore, Model 6 could be used for prediction analysis. The specific expression of Model 6 was SARIMA((1,2,3,4,6,7,11),2,1)(1,0,1)_12_.

**Table 1:** The ADF test results of original monthly electric energy production data and its secondary differential data in Xinjiang.

			t-Statistic	p-value
Original data	Augmented Dickey–Fuller test statistic		1.98	0.9999
	Test critical values	1% level	− 3.46	
		5% level	− 2.87	
		10% level	− 2.57	
Data after difference	Augmented Dickey–Fuller test statistic		− 13.48	< 0.001
	Test critical values	1% level	− 3.46	
		5% level	− 2.87	
		10% level	− 2.57

**Table 2:** Six models with their R^2^, AIC and SC passed parametric test.

	Variable	Coefficient	p-value	**R**^**2**^	**AIC**	**SC**
Model 1	AR(1)	− 0.63	< 0.001	0.8	7.03	7.12
	AR(2)	− 0.33	< 0.001			
	AR(3)	− 0.27	< 0.001			
	SAR(12)	0.92	< 0.001			
	MA(1)	− 1.06	< 0.001			
	SMA(12)	− 0.39	0.0004			
Model 2	AR(1)	− 0.59	< 0.001	0.796	7.07	7.18
	AR(2)	− 0.43	< 0.001			
	AR(3)	− 0.23	0.0033			
	AR(4)	− 0.17	0.0178			
	SAR(12)	0.92	< 0.001			
	MA(1)	− 0.99	< 0.001			
	SMA(12)	− 0.35	0.0011			
Model 3	AR(1)	− 0.57	< 0.001	0.801	7.06	7.18
	AR(2)	− 0.43	< 0.001			
	AR(3)	− 0.23	0.0035			
	AR(4)	− 0.19	0.0094			
	AR(6)	− 0.15	0.018			
	SAR(12)	0.92	< 0.001			
	MA(1)	− 0.99	< 0.001			
	SMA(12)	− 0.39	0.0007			
Model 4	AR(1)	− 0.62	< 0.001	0.799	7.07	7.2
	AR(2)	− 0.422	< 0.001			
	AR(3)	− 0.25	0.0016			
	AR(4)	− 0.16	0.0286			
	AR(7)	− 0.12	0.0347			
	SAR(12)	0.92	< 0.001			
	MA(1)	− 0.99	< 0.001			
	SMA(12)	− 0.34	0.0005			
Model 5	AR(1)	− 0.59	< 0.001	0.806	7.05	7.2
	AR(2)	− 0.45	< 0.001			
	AR(3)	− 0.27	0.0004			
	AR(4)	− 0.19	0.0071			
	AR(6)	− 0.19	0.0041			
	AR(7)	− 0.16	0.0097			
	SAR(12)	0.83	< 0.001			
	MA(1)	− 0.99	< 0.001			
	SMA(12)	− 0.25	0.0169			
Model 6	AR(1)	− 0.61	< 0.001	0.813	7.04	7.2
	AR(2)	− 0.44	< 0.001			
	AR(3)	− 0.29	0.0002			
	AR(4)	− 0.21	0.0043			
	AR(6)	− 0.221	0.0011			
	AR(7)	− 0.16	0.012			
	AR(11)	0.17	0.0181			
	SAR(12)	0.90	< 0.001			
	MA(1)	− 0.99	< 0.001			
	SMA(12)	− 0.30	0.0048

### Modeling analysis of Holt-Winters models

**Figure 6:**
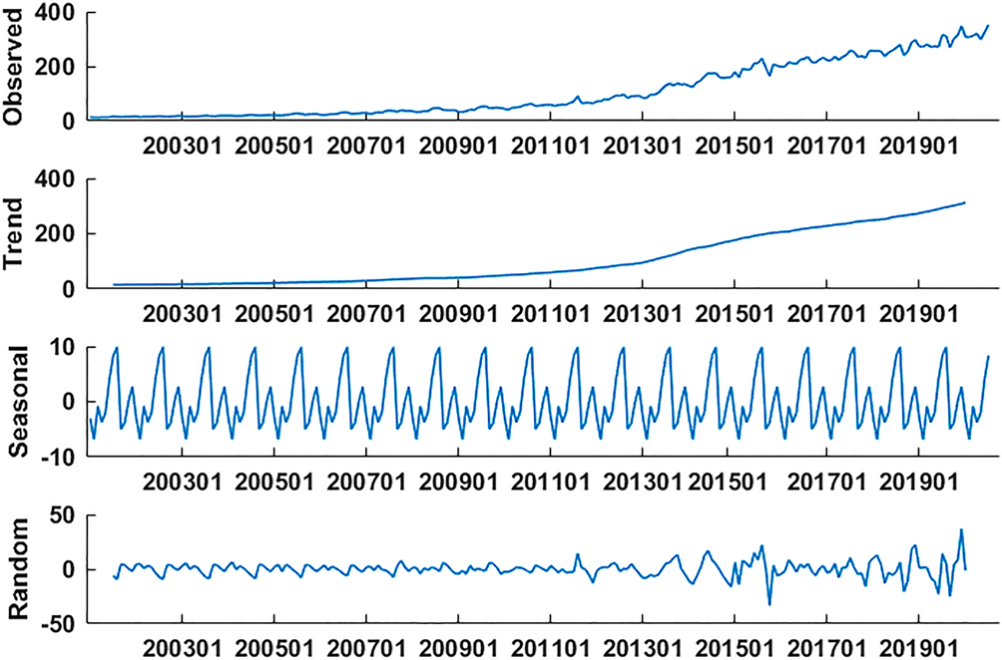
Decomposition of monthly electric energy production time series in Xinjiang from January 2001 to July 2020 (since the x-axis length is limited, only the January location of each year is marked in the figure).

**Figure 7:**
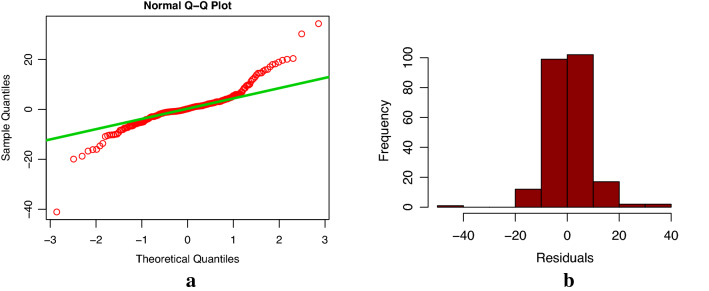
Normal distribution Q-Q graph (**a**) and histogram (**b**) of Holt-Winters' additive model residuals.

**Figure 8:**
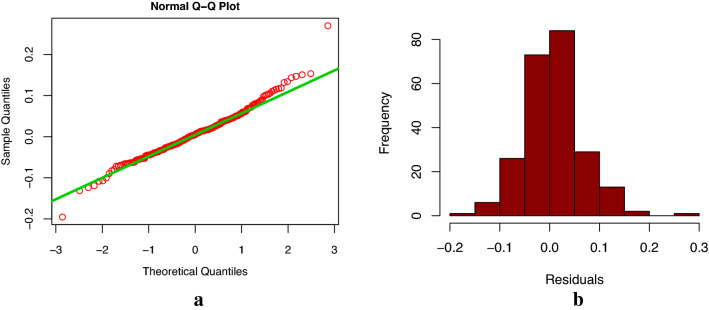
Normal distribution Q-Q graph (**a**) and histogram (**b**) of Holt-Winters' multiplicative model residuals.

According to Fig. 3, we can see that the time series for the modeling has obvious trend, and the fluctuation of data is increased with the passage of time. We decomposed the time series of Xinjiang electric energy production data from January 2001 to July 2020 using the R software decompose() function. As shown in Fig. 6, we could see that the time series was trend, seasonal and random. According to all the above data characteristics, we wanted to build the best Holt-Winters model to forecast and analyze the electric energy production data in Xinjiang. We used the ets() function package of R software to find the best smoothing parameters of model. First, we constructed Holt-Winters' additive model, we obtained $\alpha$ = 0.2418, $\beta$ = 0.0191, and $\gamma$ = 0.4914. Using the Box-Jenkins Q method to test whether the model residuals were white noise, the results showed that the p-value was less than 0.05 (p-value = 0.02). Furthermore, from the residual normal distribution Q-Q chart and histogram (see Fig. 7), we could see that the residual error did not obey the normal distribution, which indicated that the model residual was not white noise, indicating that the model fitting accuracy was not high, and the model couldn’t be used to predict Xinjiang monthly electric energy production. Second, we constructed Holt-Winters' multiplicative model, we obtained $\alpha$ = 0.6204, $\beta$ = 0.0223, and $\gamma$ = 0.0001. The p-value of Box-Jenkins Q test of model residual was more than 0.05 (p-value = 0.66) for the established multiplicative model, and the residual normal distribution Q-Q chart and histogram (see Fig. 8) showed that the residual error obeyed the normal distribution. These indicated that the residuals of Holt-Winters' multiplicative model was white noise, and fitting accuracy of this model was high. Therefore, Holt-Winters' multiplicative model could be used to predict Xinjiang monthly electric energy production.

### Model comparison

**Figure 9:**
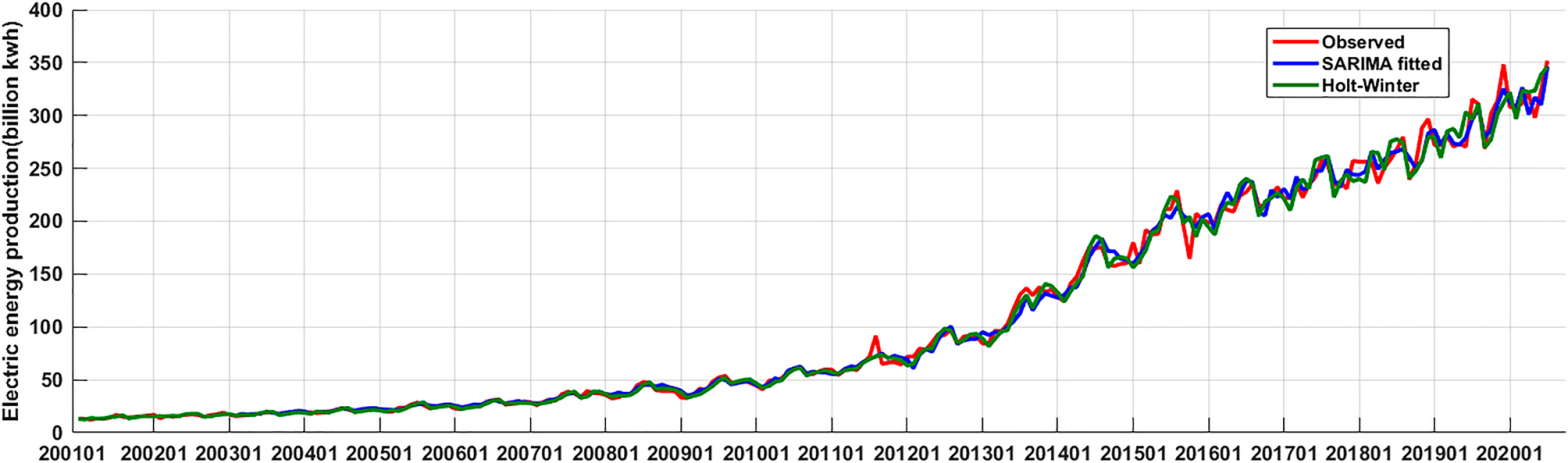
The comparison of fitting effects of the SARIMA((1,2,3,4,6,7,11),2,1)(1,0,1)12 model and the Holt-Winters' multiplicative model (since the x-axis length is limited, only the January location of each year is marked in the figure).

**Figure 10:**
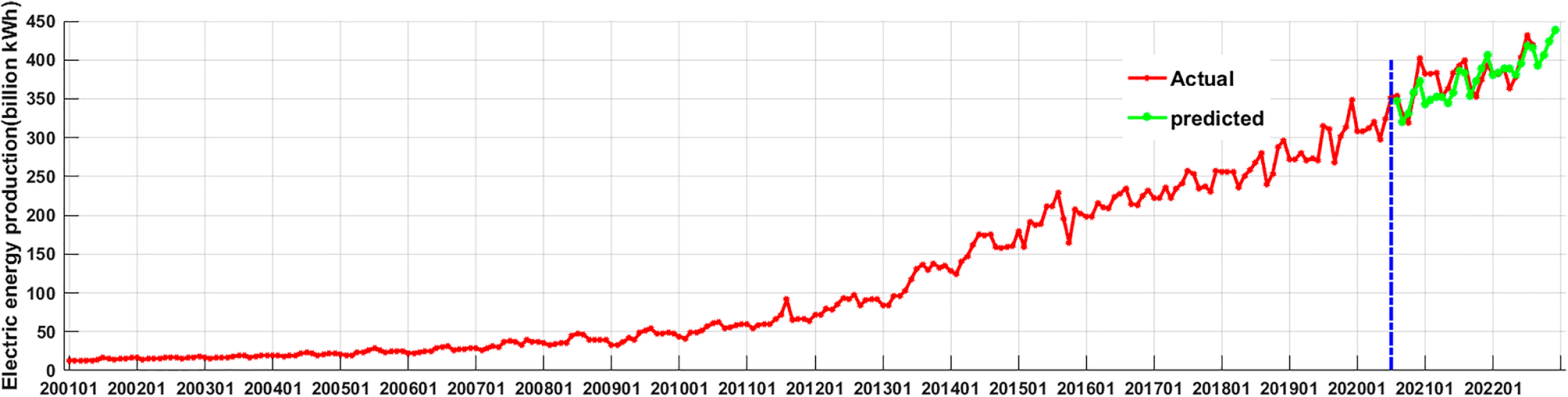
The curves of actual values and predicted values of electric energy production of Xinjiang (since the x-axis length is limited, only the January location of each year is marked in the figure).

Both the SARIMA((1,2,3,4,6,7,11),2,1)(1,0,1)_12_ model and the Holt-Winters' multiplicative model could fit Xinjiang power generation time series well, we calculated the fitting precision indexes RMSE, MAE and MAPE of two models respectively (see Table 3). Based on these two models, we predicted the monthly electric energy production in Xinjiang from August 2020 to July 2021, and calculated the prediction precision indexes RMSE, MAE and MAPE of two models respectively (see Table 3). The fitting and prediction performance of the two models was compared by these index values in Table 3. The smaller the three index values, the better the performance of model. The comparison showed that there was little difference in fitting ability between the two models. The RMSE of the SARIMA((1,2,3,4,6,7,11),2,1)(1,0,1)_12_ model was less than that of Holt-Winters' multiplicative mode, but the MAE and MAPE of Holt-Winters' multiplicative model were less than that of SARIMA((1,2,3,4,6,7,11),2,1)(1,0,1)_12_ model. For a more intuitive comparison, we drew Fig. 9. From Fig. 9, we could see that the two models had almost the same fitting ability, and their fitting precisions were both very high. However, as can be seen from the predictive accuracy indexes, the three accuracy indexes of the SARIMA((1,2,3,4,6,7,11),2,1)(1,0,1)_12_ model are significantly smaller than that of the Holt-Winters' multiplicative model, so, overall, the SARIMA((1,2,3,4,6,7,11),2,1)(1,0,1)_12_ model performs better. Therefore, we considered using the SARIMA (1,2,3,4,6,7,11),2,1)(1,0,1)_12_ model to predict Xinjiang's monthly electric energy production from August 2021 to December 2022. The forecast results were shown in Table 4 and Fig. 10.

**Table 3:** The fitting and prediction accuracy values of SARIMA((1,2,3,4,6,7,11),2,1)(1,0,1)_12_ model and Holt-Winters' multiplicative model.

Indexes	Fitting			Prediction		
	RMSE	MAE	MAPE	RMSE	MAE	MAPE
SARIMA((1,2,3,4,6,7,11),2,1)(1,0,1)_12_	**7.8**	**5.14**	**4.87**	**22.09**	**17.96**	**4.78**
Holt-Winter multiplicative model	**8.34**	**4.79**	**4.3**	**48.77**	**40.76**	**10.82**

**Table 4:** The actual values and the predicted values by the SARIMA((1,2,3,4,6,7,11),2,1)(1,0,1)12 model of monthly electric energy production (billion kWh) of Xinjiang.

Date	SARIMA Prediction	Actual	Error	Date	SARIMA Prediction	Actual	Error
202108	383.16	399.50	16.3	202205	380.49	378.20	− 2.29
202109	353.68	364.30	10.62	202206	395.28	403.50	8.22
202110	372.3	353.00	− 19.3	202207	418.57	431.60	13.03
202111	388.95	374.70	− 14.25	202208	416.38	419.60	3.22
202112	406.09	393.50	− 12.59	202209	392.35		
202201	381.05	381.70	0.65	202210	406.54		
202202	383.09	381.70	− 1.39	202211	423.78		
202203	388.65	388.00	− 0.65	202212	438.99		
202204	388.98	362.90	− 26.08				

Error = Actual − SARIMA prediction.

## Discussion

In this study, firstly, according to the characteristics of Xinjiang monthly electric energy production time series data, we established the best SARIMA((1,2,3,4,6,7,11),2,1)(1,0,1)_12_ model. We could see from Table 2 (model 6) that all the parameters of the model passed the test (p-value were less than 0.05). From the autocorrelation and partial correlation Fig. 5 of the model residuals, it can be seen that the autocorrelation and partial correlation coefficients of the SARIMA((1,2,3,4,6,7,11),2,1)(1,0,1)_12_ model residuals were basically in the double standard deviation, indicating that the residuals of the SARIMA((1,2,3,4,6,7,11),2,1)(1,0,1)_12_ model were white noise, and the model had good performance. We could see from the fitting curve of the historical data of the SARIMA((1,2,3,4,6,7,11),2,1)(1,0,1)_12_ model (in Fig. 9) that the fitting curve of the model basically coincided with the original Xinjiang monthly electric energy production time series, which indicated that the fitting accuracy of the model was very high. Secondly, we used the ets() function package of R software to construct Holt-Winters' additive model, but when we did the residual test of the model, the result showed that the model residuals were not white noise, therefore, the model fitting accuracy was not high, and Holt-Winters' additive model was not suitable for predicting the future monthly electric energy production of Xinjiang. Finally, we constructed the Holt-Winters' multiplicative model, the p-value of the residual test of the model was greater than 0.05 and the Q-Q chart of model residuals and Histogram (see Fig. 8) showed that the residuals basically obeyed normal distribution, which indicated that the model residuals were white noise and the model had good performance. Using Holt-Winters' multiplicative model to fit the historical data of Xinjiang monthly electric energy production (see Fig. 9), the model fitting curve basically coincided with the original Xinjiang monthly electric power output time series, which indicated that the model fitting accuracy was very high. To establish the best forecast model of Xinjiang monthly electric energy production, we compared the SARIMA((1,2,3,4,6,7,11),2,1)(1,0,1)_12_ model and Holt-Winters' multiplicative model fitting accuracy and prediction accuracy (see Table 3). We found that the SARIMA((1,2,3,4,6,7,11),2,1)(1,0,1)_12_ model has a better predictive performance than that of the Holt-Winters' multiplicative model. Therefore, we applied the SARIMA((1,2,3,4,6,7,11),2,1)(1,0,1)_12_ model to predict Xinjiang's monthly electric energy production from August 2021 to December 2022. From Table 4, we can see the errors are relatively small, which indicates SARIMA((1,2,3,4,6,7,11),2,1)(1,0,1)_12_ model can well predict the electric energy production in Xinjiang. From the Fig. 10 we can see that the monthly electric energy production of Xinjiang from August 2020 to December 2022 shows a fluctuating upward trend, which is consistent with the actual situation.

Some studies often found that the prediction effect of a single model was not good, so the combination prediction was used, and their research results showed that the combination prediction could achieve more accurate results^35^. However, in this case, the prediction model is often more complex and not easy to operate in the actual prediction analysis. In our study, three models were used, and two models were compared for the prediction performance. A series of analysis results showed that the single SARIMA((1,2,3,4,6,7,11),2,1)(1,0,1)_12_ model has high prediction accuracy when predicting the output of Xinjiang electric power (see Fig. 9). A single model is relatively simple and is easier to use when doing the actual predictive analysis.

In this study, although the fit and prediction accuracy of the SARIMA((1,2,3,4,6,7,11),2,1)(1,0,1)_12_ model are relatively high, there are also some errors. The reason of errors is that there are many factors affecting electricity production, such as population size, industrial development scale, people's living standards, the speed of economic development, and public health emergencies such as COVID-19. In our study, we only used historical power production data for predictive analysis, not considering other factors, because we thought that adding these factors will increase the model complexity, and these factors will also have many uncertainties, which may not necessarily improve the prediction accuracy of the model. Interested readers can do further research.

Considering that the forecasting uncertainty may affect the decision making process and increase the risk of scheduling, some studies have used interval prediction for their predictive analysis and got better prediction effect^36,37^. In our next-step study, we will consider doing interval prediction analysis in an attempt to find models with higher optimal prediction accuracy.

## Conclusions

Electric Power plays a vital role in the national economy and people's livelihood, especially in the peak period of electricity consumption. Early prediction of electric energy production can provide scientific reference for the rational planning and distribution of power demand. Based on the monthly power output data of Xinjiang from January 2001 to August 2022, this study is the first time to construct a prediction model that can relatively accurately predict the electric energy production in Xinjiang. Although the methods we used were not complex, our prediction accuracy was very high, which provided a kinds of simple and easy-to-use scientific tools for the future energy production prediction in Xinjiang. Our research methods and research ideas can also provide a reference for other researchers to make power prediction in some place.

## Data Availability

The datasets used and/or analysed during the current study available from the corresponding author on reasonable request.
